# Case Report: Anlotinib for advanced chordoma

**DOI:** 10.3389/fonc.2026.1760330

**Published:** 2026-01-15

**Authors:** Zhining Jing, Chunmiao Song, Yongqiang Jiang

**Affiliations:** Department of Neurosurgery, Baotou Central Hospital, Affiliated Baotou Clinical College of inner Mongolia Medical University, Baotou, Inner Mongolia, China

**Keywords:** advanced cancer, anlotinib, case report, chordoma, molecular targeted drugs

## Abstract

Chordoma is a rare tumor with low to moderate malignancy. It is typically treated with surgical resection in the early stages. However, due to the slow growth and locally invasive biological characteristics of chordoma, patients are often diagnosed at an advanced stage, rendering them ineligible for surgery. This case report describes a 71-year-old male patient who experienced notable symptom relief after receiving single-agent anlotinib treatment for advanced thoracic chordoma. According to the World Health Organization (WHO) criteria for solid tumors, imaging examinations indicated that the patient achieved a partial response (PR). These findings suggest that anlotinib has potential therapeutic value in the treatment of chordoma, primarily in managing symptoms and tumor progression. In this report, we retrospectively analyzed the patient’s case data and discussed it in conjunction with relevant literature to provide new therapeutic strategies for clinicians facing similar patients.

## 1 Introduction

Chordoma is a rare malignant bone tumor that originates from embryonic remnants of notochordal tissue (1). It typically occurs in midline structures of the spine, including the sacrococcygeal region (50%-60%), the skull base (25%-35%), and the vertebral column (15%-20%) (2). The clinical manifestations of chordoma are diverse, with common symptoms including localized pain, mass effect, and neurological dysfunction. The rate of distant metastasis in chordoma is relatively low; however, once metastasis occurs, multiple organs are often involved. Surgery is the cornerstone of chordoma treatment (3). Due to the complex anatomy, complete resection of the tumor is often challenging, leading to a high recurrence rate. Patients with advanced disease are prone to distant metastasis. Chordoma shows strong resistance to conventional chemotherapy and radiotherapy, and the efficacy of chemotherapy and radiotherapy as adjuvant therapy post-surgery is limited, resulting in poor prognosis for patients.

Molecular targeted therapy is a candidate therapeutic approach for advanced malignant tumors. Anlotinib is a multi-target tyrosine kinase inhibitor that exerts its anti-tumor effects primarily by inhibiting the vascular endothelial growth factor receptors (VEGFR), including other related signaling pathways (4). In recent years, anlotinib has demonstrated good efficacy in the treatment of various malignant tumors, particularly in diseases such as non-small cell lung cancer, and hepatocellular carcinoma (5–7). Although research on anlotinib in the treatment of chordoma is still limited, preliminary evidence suggests potential efficacy of anlotinib in chordoma treatment (8). In this study, we present a case of advanced chordoma with significant symptom relief following treatment with anlotinib and provide a comprehensive overview of current treatment strategies for chordoma.

## 2 Case description

**Figure 1:**
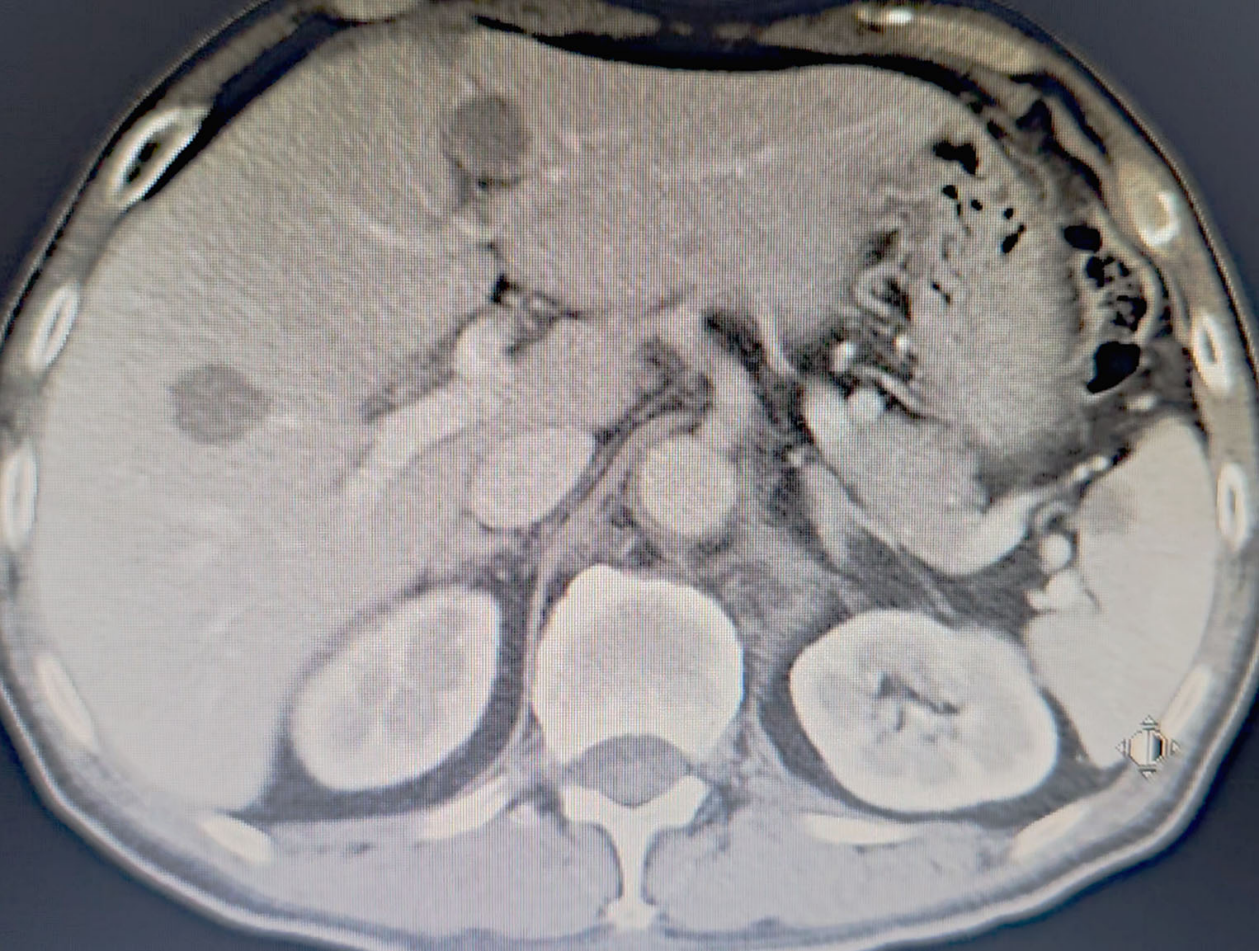
Two obvious low-density round shadows in the liver.

**Figure 2:**
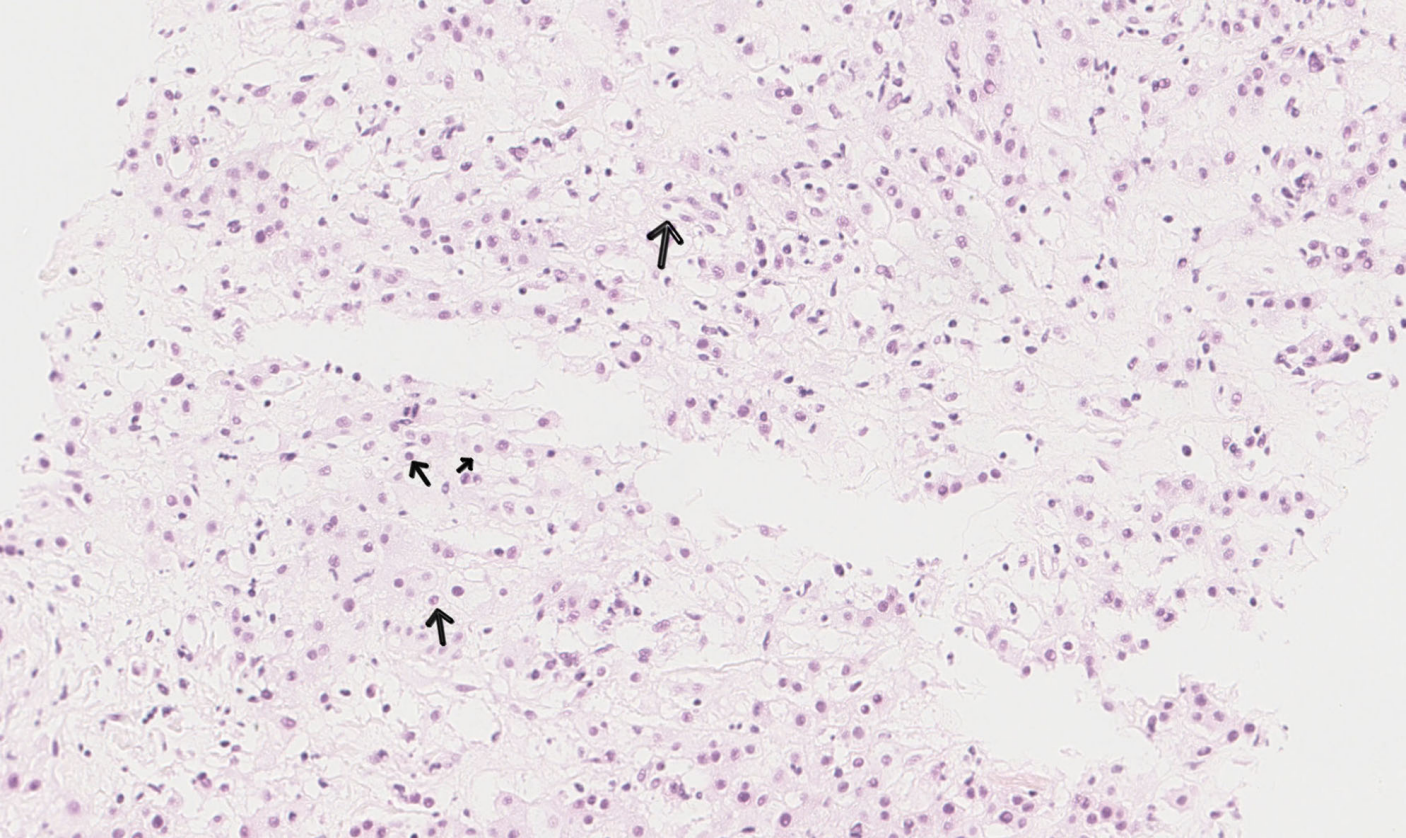
Pathological smear slide shows epithelial-like cells, eosinophilic cytoplasm, and mucinous stroma; the arrow indicates vacuolated cells.

A 71-year-old male patient visited Baotou Steel Hospital in December 2022 due to “intermittent abdominal pain for one month, with symptoms gradually worsening.” A CT scan revealed possible metastatic lesions in the liver, right kidney, and the 11th thoracic vertebra and surrounding soft tissue (**Figure 1**). Subsequently, a puncture biopsy of the abdominal mass was performed at our hospital. Pathological findings: Microscopic examination revealed epithelioid tumor cells arranged in glandular and cord-like patterns within a fibrous stroma. The cells exhibited hyperchromatic nuclei with mild atypia (**Figure 2**). Immunohistochemical staining demonstrated that the tumor cells were positive for pan-cytokeratin (CKpan), Vimentin, Synaptophysin (Syn), CK8, CK18, CD56 (focal), and EGFR. The Ki-67 proliferation index was approximately 40%. Results were negative for TTF-1, NapsinA, CK7, CK20, Chromogranin A (CgA), PAX8, CD10, CD68, p63, and RCC. p53 showed a wild-type staining pattern. For further confirmation, the biopsy specimens were reviewed at Peking University Third Hospital. Additional immunohistochemistry revealed strong nuclear positivity for Brachyury, retained expression of INI-1, and negativity for Glypican-3, CD34, and Inhibin-α. CD31 staining was only weakly positive. Final Diagnosis: Based on the combined morphological and immunohistochemical profile, notably the positivity for Brachyury, the patient was diagnosed with chordoma of the 11th thoracic vertebra (T11) with multiple metastases to the liver and right kidney.

**Figure 3:**
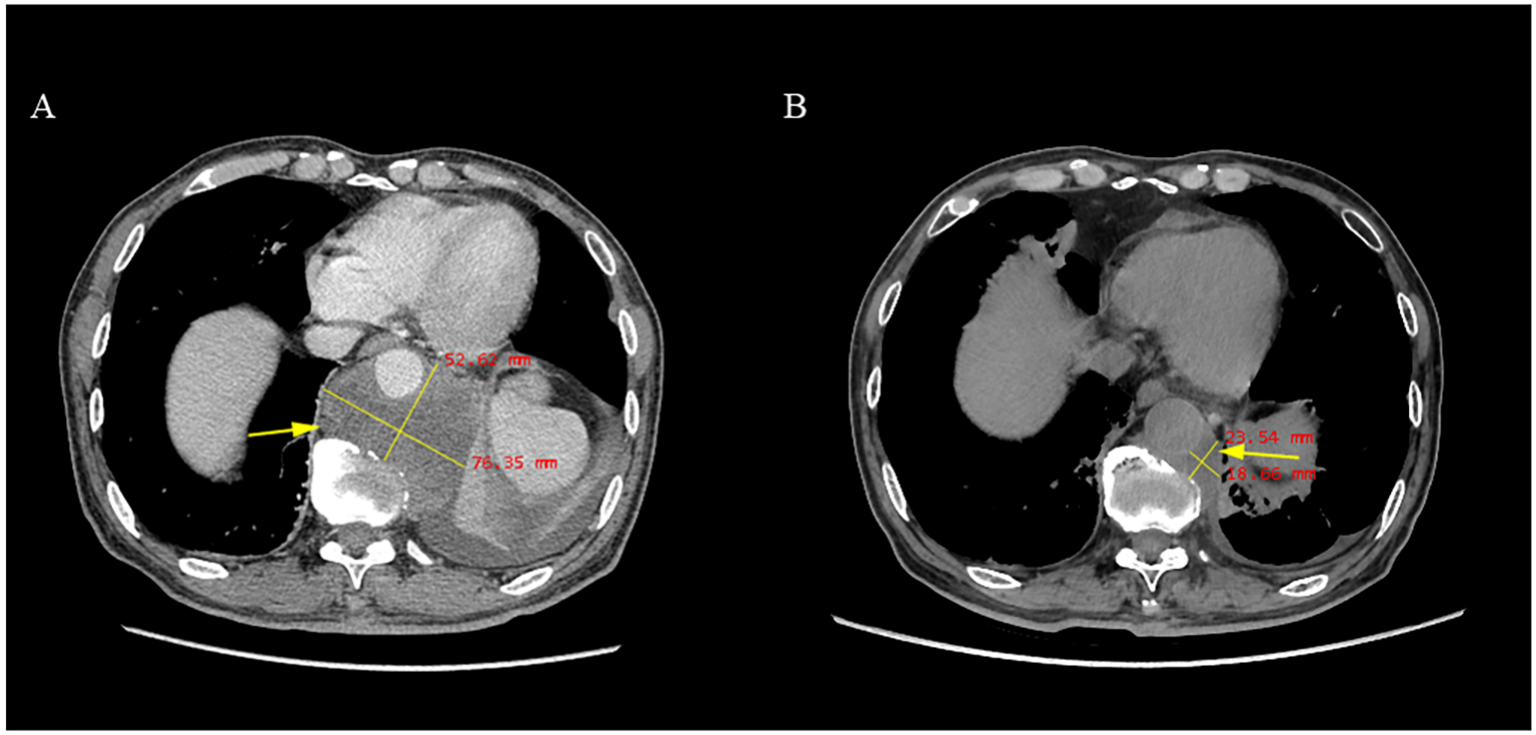
**(A)** Imaging before anlotinib treatment showed a tumor with a major diameter of 76.35 mm, completely encircling the thoracic aorta; **(B)** Imaging four months after anlotinib treatment showed a tumor with a major diameter of 23.54 mm, not completely encircling the thoracic aorta (the measurement positions for both tests were at the T11 level cross-section).

**Figure 4:**
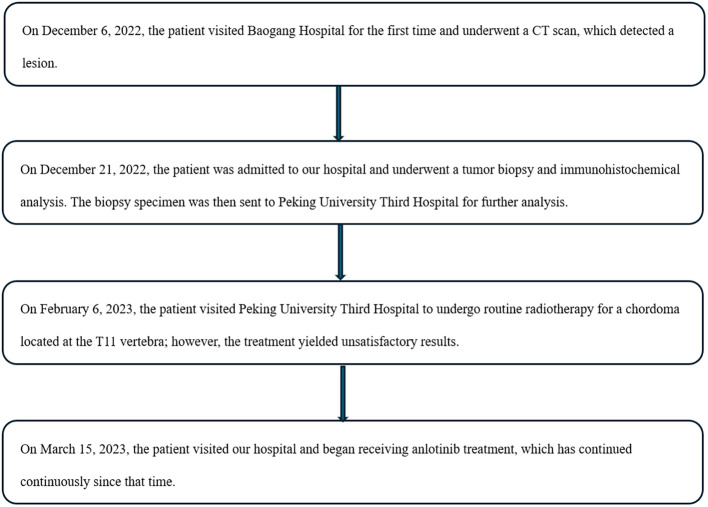
Timeline of this case.

The patient underwent conventional radiotherapy at Peking University Third Hospital on February 6, 2023, but clinical symptoms did not significantly improve. Subsequently, the patient visited our hospital in March 2023. Considering the diagnosis of a special type of advanced chordoma with multiple systemic metastases, oral treatment with anlotinib was initiated. The specific treatment regimen is a 21-day cycle, taking 12 mg orally daily for 2 weeks, followed by a 1-week drug holiday, and the regimen continues to date. Currently, the patient’s pain symptoms have improved. Aside from a history of hypertension, no complications such as nausea, vomiting, diarrhea, or hand-foot syndrome were observed. The patient underwent regular follow-ups, with no significant adverse reactions noted, demonstrating good drug tolerance. (Adverse events related to treatment are assessed according to the Common Terminology Criteria for Adverse Events (CTCAE) version 5.0 by the National Cancer Institute.)The most recent CT follow-up indicated that the tumor had reduced by more than 50%. According to the WHO criteria for solid tumors, the patient has now achieved PR (**Figure 3**). The timeline of this case is depicted in **Figure 4**.

## 3 Discussion

Chordoma is a rare malignant tumor characterized by strong local invasiveness, high recurrence rates, and a lack of effective systemic treatment options. Clinical management of chordoma is particularly challenging when the tumor is located at the skull base and spine, leading to a heavy disease burden (9). Epidemiological studies indicate that the incidence of chordoma is approximately 0.08 per 100,000 individuals. The incidence is higher in people around 60 years of age and is more common in males, with a male-to-female ratio of approximately 2:1. In contrast, the incidence in children is low (9, 10). Anatomically, chordoma can originate from any part along the embryonic notochord, with comparable proportions found in the sacrum (29.2%), spine (32.8%), and skull base (32%) (9). Histologically, the World Health Organization classifies chordomas into three types: conventional chordoma, dedifferentiated chordoma, and poorly differentiated chordoma. Conventional chordoma is the most common type, accounting for approximately 90% of cases (11). The cases discussed in this article also belong to this type. Despite the continuous evolution of treatment strategies, the 5-year survival rate for chordoma remains relatively low, significantly influenced by tumor location, treatment methods, and patient characteristics (12, 13).

In recent years, scholars have classified chordoma into bone microenvironment-dominant subtype, mesenchymal-derived subtype, and mesenchymal-to-epithelial transition (MET)-mediated subtypes based on proteomics and gene expression profiling (14). Early clinical manifestations of chordoma are often subtle, and it is typically discovered at an advanced stage. Different tumor locations and their compression of surrounding tissues exhibit different clinical symptoms: sacrococcygeal chordoma often presents with localized pain, masses, constipation, and urinary incontinence; skull base chordoma presents with headaches, visual disturbances, diplopia, and dysphagia; spinal chordoma presents with back pain, radicular pain, and spinal cord compression symptoms (such as limb weakness and sensory abnormalities). If distant metastasis occurs (such as to the lungs, liver, or kidneys), it may lead to cough, dyspnea, abdominal pain, and other symptoms. In this case, the patient’s primary lesion at thoracic vertebra 11 (T11) did not exhibit neurological compression symptoms but rather presented with abdominal pain due to distant metastasis.

The diagnosis of chordoma requires a comprehensive approach using imaging, pathology, and clinical examinations. Magnetic resonance imaging (MRI) is the preferred imaging method, clearly showing the extent of the tumor and its relationship with surrounding structures. Computed tomography (CT) can clearly display bone destruction and tumor calcification, especially due to its high resolution for bony structures at the skull base and spine. Positron emission tomography-computed tomography (PET-CT) is used to assess tumor metabolic activity and distant metastasis, particularly suitable for recurrent or metastatic chordoma. Pathological examination is the gold standard for diagnosing chordoma (15); tissue samples are typically obtained through biopsy or surgical resection, and the immunohistochemical marker Brachyury is highly specific for chordoma diagnosis (16). Through comprehensive diagnosis, optimal treatment plans can be formulated for patients with chordoma to maximize treatment efficacy and minimize complications. However, in this case, the patient refused PET-CT examination due to financial reasons.

Currently, the first-line treatment methods for chordoma remain controversial (17), although surgical resection is one of the main treatment modalities. Surgical approaches typically include the following: 1. Wide en bloc excision. This aims to completely remove the tumor without violating the capsule, ensuring negative margins. 2. Intralesional resection. This refers to piecemeal removal of the tumor and capsule, aiming to achieve negative margins. 3. Planned debulking surgery. This involves removing part of the tumor to improve neurological deficits and relieve pain, without aiming for negative margins (18, 19). Scholars generally believe that wide en bloc excision during the initial surgery provides the best treatment option and may improve patient prognosis and survival rates. However, in most cases, complete resection of the tumor is extremely difficult or even impossible due to the axial location of the tumor (20, 21). In this case, the patient had a massive tumor that wrapped around the abdominal aorta and was accompanied by multiple organ metastases, making complete resection not feasible.

Chordoma shows poor sensitivity to radiotherapy and chemotherapy, with limited efficacy of conventional radiotherapy and chemotherapy. Studies have reported reduced survival rates in patients with active spinal chordoma receiving radiotherapy (22, 23). In recent years, advancements in high-dose focused radiation delivery techniques, including particle therapy (protons, carbon ions) and photon therapy (stereotactic radiosurgery, intensity-modulated radiation therapy [IMRT], or fractionated stereotactic radiotherapy [SRT]), have enabled higher doses of radiation to be delivered to tumors while protecting surrounding normal tissues. For example, hypofractionated proton or photon therapy has been proposed as an effective alternative to traditional radiotherapy plans (24, 25). The 5-year overall survival rate for high-dose image-guided proton therapy can reach 85.4% (26), demonstrating significant efficacy. The application of preoperative radiotherapy in spinal chordoma and sacrococcygeal chordoma is under investigation, aiming to reduce the local recurrence rate caused by tumor dissemination during surgery. Overall, new radiation therapies have made progress in treating inoperable chordoma cases. Unfortunately, these novel radiation therapies are still in clinical trial stages, and the patient in this case was unable to receive such treatments; conventional radiotherapy shows no substantial progress in treatment. Although some individual cases have shown positive responses to cytotoxic chemotherapy (27), overall, chordoma rarely responds to systemic chemotherapy, and the responses are often short-lived, with limited long-term efficacy.

Targeted drugs are used as second-line treatments for chordoma, commonly in advanced patients who cannot undergo surgical resection or for whom radiotherapy is ineffective. Currently, there are no approved targeted therapies for chordoma, and relevant animal models are scarce; however, many preclinical drug studies have been conducted (28). In recent years, targeted therapy has made some progress, and immunotherapy has also garnered widespread attention, yielding some early positive results. Brachyury, a transcription factor of the T-box gene family (29), is highly expressed in chordoma and has high specificity, making it a potential target for targeted therapy. Previously reported targeted drugs include receptor tyrosine kinase inhibitors (TKIs), such as imatinib and dasatinib, which block tumor cell proliferation signaling pathways by inhibiting platelet-derived growth factor receptor (PDGFR) and KIT receptor tyrosine kinase activitypidermal growth factor receptor (EGFR) and human epidermal growth factor receptor 2 (HER2) inhibitors such as erlotinib, lapatinib, gefitinib, and cetuximab inhibit EGFR and HER2 activity, thereby suppressing tumor cell growth and proliferation. Anti-angiogenic drugs such as vascular endothelial growth factor receptor (VEGFR) inhibitors sorafenib, pazopanib, and sunitinib reduce tumor angiogenesis by inhibiting VEGFR activity, limiting tumor nutrient supply and growth. Mechanistic target of rapamycin (mTOR) inhibitors such as temsirolimus and sirolimus primarily inhibit mTOR complex 1 (mTORC1) activity, blocking tumor cell growth and proliferation signaling pathways.

In one study, Yin et al. first revealed the clinical and proteomic characteristics of chordoma based on a large cohort. They further proposed specific therapeutic interventions targeting molecular subtypes. The study emphasized that the MET-mediated chordoma subtype might respond well to TKIs, and anlotinib shows promise as a potentially optimized treatment option for this subtype (14). Notably, according to currently published literature, there are no clinical reports of anlotinib used for the treatment of chordoma; therefore, this study is the first to report the application of anlotinib in chordoma treatment. The favorable response observed in our case provides a clinical observation that is hypothesis-generating and compatible with the preclinical findings of Yin et al., who suggested MET-mediated subtypes might be sensitive to TKIs. Future prospective studies incorporating tumor molecular profiling are warranted to validate this association.

## 4 Limitations

This study has several limitations. First, as a single case report (N-of-1 design), its results cannot be generalized to a broader chordoma population and lack the statistical power of a controlled trial. Second, we did not conduct a comprehensive molecular profiling analysis of the tumor, such as genomic sequencing. Therefore, we cannot confirm the biological association between the observed efficacy of anlotinib and specific molecular subtypes, such as the MET-mediated subtype; this finding is merely hypothesis-generating. Third, the current follow-up time is still relatively short, and the long-term efficacy, duration of response, and potential resistance to anlotinib remain unknown. Finally, in the absence of a control group, it is impossible to completely rule out the influence of other unmeasured factors or concomitant treatments on the patient’s condition.

## 5 Conclusion

In summary, chordoma is a rare malignant tumor with a poor prognosis. Contrast-enhanced CT, and MRI are valuable diagnostic tools. Currently, the only potentially curative treatment is complete resection with negative surgical margins (R0). The effectiveness of radiotherapy and chemotherapy for chordoma remains uncertain, while targeted therapy may emerge as a novel treatment option based on ongoing research. Regular monitoring, early detection, and prompt treatment are key to improving quality of life, and extending survival for patients with chordoma. Given that this patient’s tumor is located in an anatomically challenging site and carries a high risk of recurrence, we will continue to closely monitor the patient and implement appropriate adjuvant therapy as needed to prolong survival.

## Data Availability

The original contributions presented in the study are included in the article/supplementary material. Further inquiries can be directed to the corresponding author.
